# Supplementary material for: Role of the nuclear membrane protein Emerin in front-rear polarity of the nucleus
Source: Nat Commun. 2020 May 1;11:2122. doi: 10.1038/s41467-020-15910-9 (PMC7195445; doi:10.1038/s41467-020-15910-9)
Supplement: Supplementary file 1 — Supplementary Information [file 41467_2020_15910_MOESM1_ESM.pdf]

Supplementary Information for

Role of the nuclear membrane protein Emerin in front-rear polarity of the nucleus

Paulina Nastały, Divya Purushothaman, Stefano Marchesi, Alessandro Poli, Tobias Lendenmann, Gururaj Rao Kidiyoor, Galina Vladimirovna Beznoussenko , Stefania Lavore, Orso Maria Romano, Dimos Poulikakos, Marco Cosentino Lagomarsino, Alexander Mironov, Aldo Ferrari, Paolo Maiuri

Correspondence to: paolo.maiuri@ifom.eu

This file includes:

Supplementary Figure 1
Supplementary Figure 2
Supplementary Figure 3
Supplementary Figure 4
Supplementary Figure 5
Supplementary Figure 6
Supplementary Figure 7
Supplementary Text
Supplementary Figure 8
Supplementary Figure 9
Supplementary Figure 10
Supplementary Figure 11
Supplementary Table 1

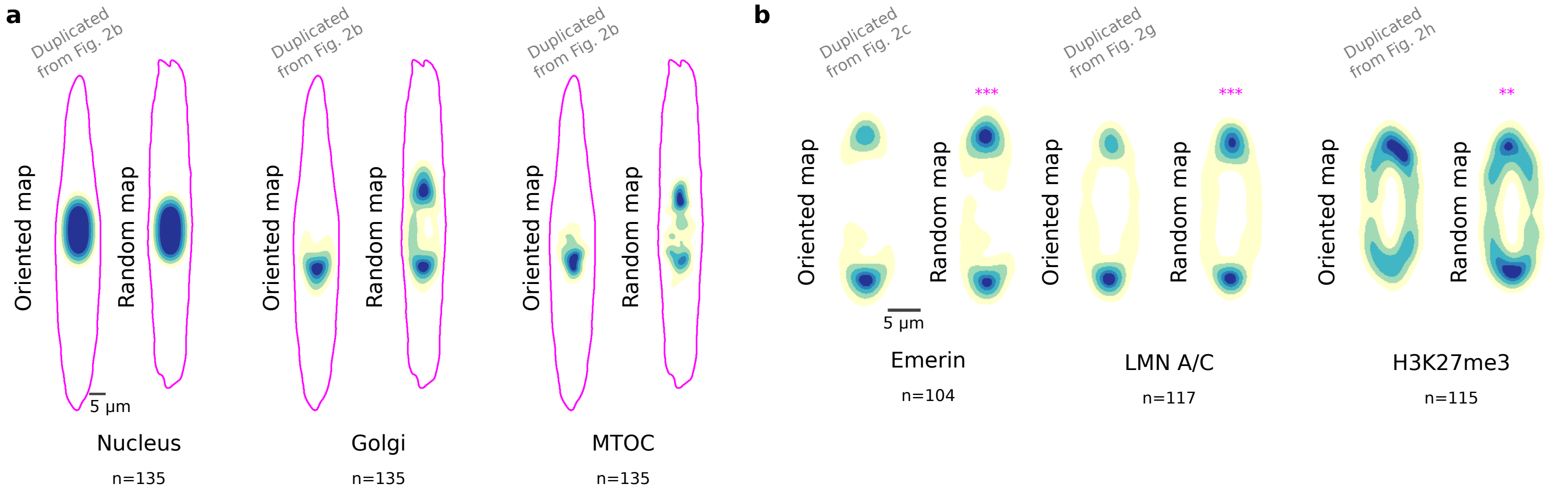
a
Duplicated from Fig. 2b
Oriented map
Random map
5 µm
Nucleus
n=135
Golgi
n=135
MTOC
n=135
b
Duplicated from Fig. 2c
Duplicated from Fig. 2g
Duplicated from Fig. 2h
***
**
5 µm
Emerin
n=104
LMN A/C
n=117
H3K27me3
n=115

Supplementary Figure 1 Random maps of organelle and protein distribution.

a Oriented versus random maps for nucleus (left), Golgi apparatus (middle) and MTOC (right), outline represents F-actin border of the averaged cells. **b** Oriented versus random maps for selected proteins. P_{EMD}=0.0005, P_{LMNAC}=4.7x10^{-8}, $P_{H3K27me3}$=0.0016, two-sided Cramer von Mises test. ***P<0.001 **P<0.01. For each distribution map an exact number of cells from 3 independent experiments is stated in the figure.

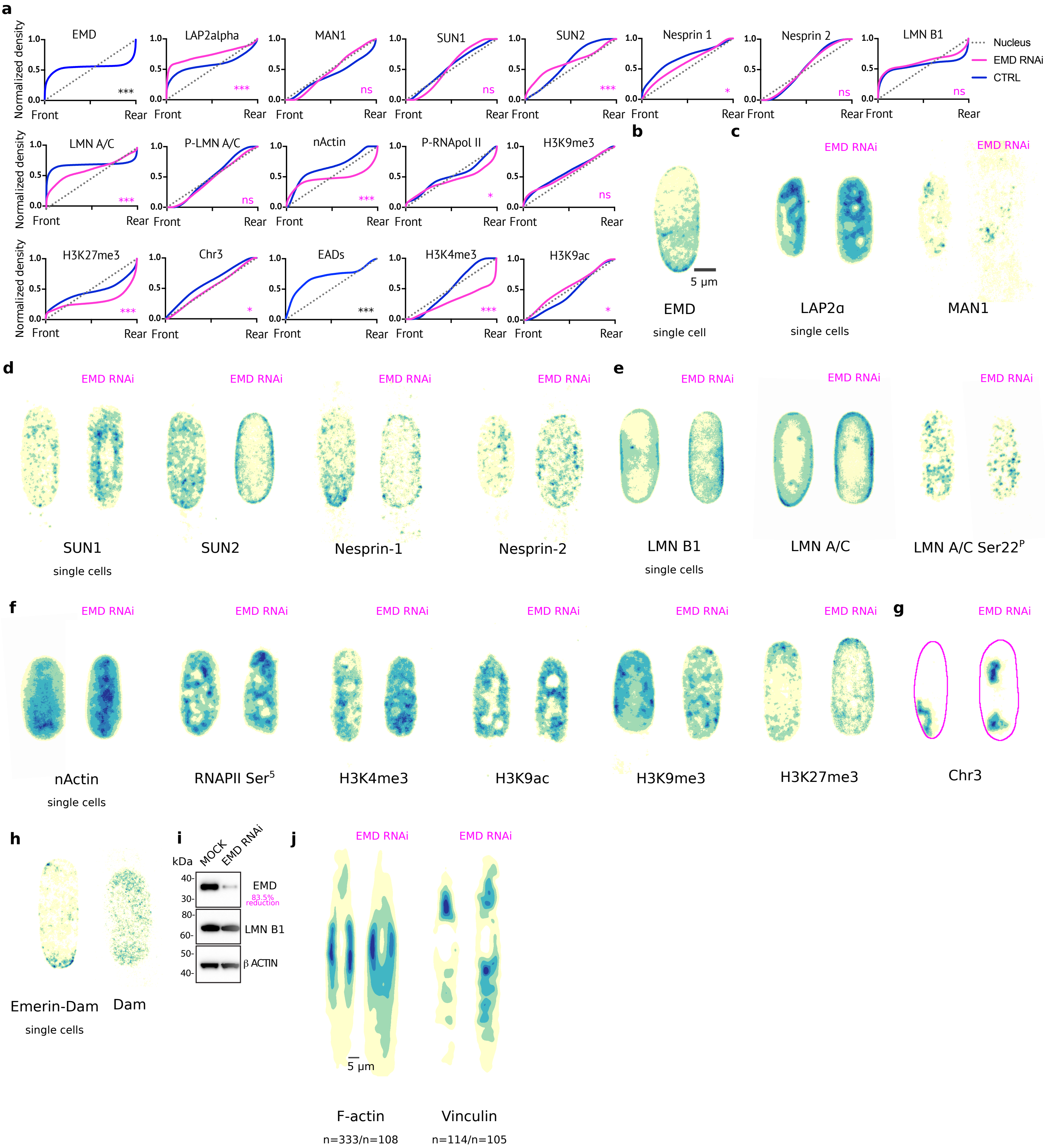

a
Normalized density
EMD
LAP2alpha
MAN1
SUN1
SUN2
Nesprin 1
Nesprin 2
LMN B1
Nucleus
EMD RNAi
CTRL
LMN A/C
P-LMN A/C
nActin
P-RNApol II
H3K9me3
H3K27me3
Chr3
EADs
H3K4me3
H3K9ac
Front
Rear
ns
b
c
EMD
single cell
5 μm
LAP2α
single cells
MAN1
d
SUN1
single cells
SUN2
Nesprin-1
Nesprin-2
e
LMN B1
single cells
LMN A/C
LMN A/C Ser22P
f
nActin
single cells
RNAPII Ser5
H3K4me3
H3K9ac
H3K9me3
H3K27me3
g
Chr3
h
Emerin-Dam
Dam
single cells
i
kDa
MOCK
EMD RNAi
EMD
83.5% reduction
LMN B1
β ACTIN
j
F-actin
Vinculin
n=333/n=108
n=114/n=105

Supplementary Figure 2 Mapping front-rear polarity in the nucleus.

a Protein distribution plots of normalized density, corresponding to maps in Figure 2c, 2e-j. ***P<0.001 **P<0.01 *P<0.05, ns – not significant. **b** Representative image of a single cell stained for emerin. **c** Representative images of single cells stained for LEM domain containing proteins, control (left) and EMD knock-down (right). **d** Representative images of single cells stained for LINC-complex proteins, control (left) and EMD knock-down (right). **e** Representative images of single cells stained for lamins (LMN), LMN A/C Ser$_{22}$ – Ser22 phosphorylated lamin A/C, control (left) and EMD knock-down (right). **f** Representative images of single cells stained for transcription-related markers, nActin – nuclear actin, RNAPII Ser5$_{P}$ – Ser5 phosphorylated RNA polymerase II, control (left) and EMD knock-down (right). **g** Representative images of single cells after performing FISH for chromosome 3, control (left) and EMD knock-down (right), the magenta outline indicates nucleus border. **h** Representative images of single cells expressing Emerin-Dam and Dam protein alone. **i** Western blot of MOCK control and EMD RNAi performed in RPE1 cells. **j** Distribution maps of F-actin and vinculin in control (left) and EMD knock-down (right). For each distribution map an exact number of cells from 3 independent experiments is stated in the figure. Source data are provided as Source Data file.

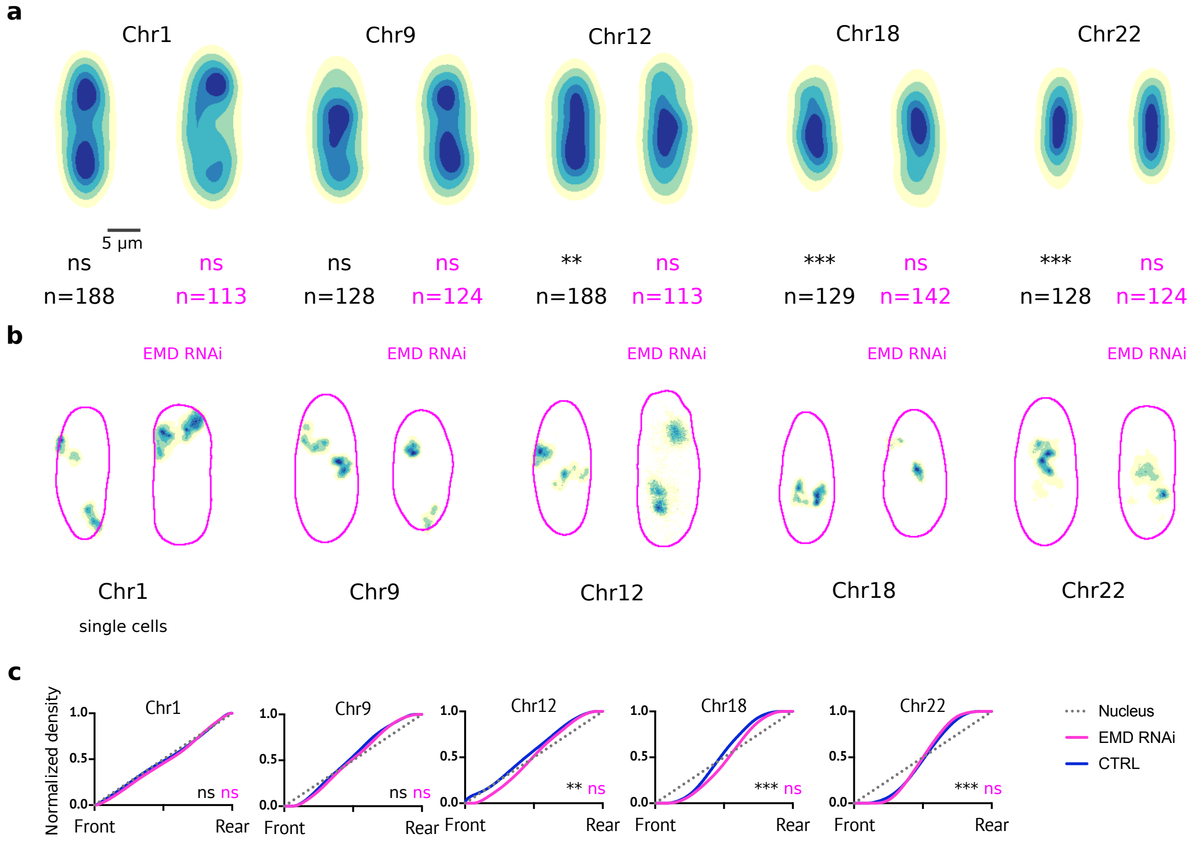
a
Chr1
Chr9
Chr12
Chr18
Chr22
5 µm
ns
ns
ns
ns
**
ns
***
ns
***
ns
n=188
n=113
n=128
n=124
n=188
n=113
n=129
n=142
n=128
n=124
b
EMD RNAi
EMD RNAi
EMD RNAi
EMD RNAi
EMD RNAi
Chr1
single cells
Chr9
Chr12
Chr18
Chr22
c
Normalized density
1.0
0.5
0.0
Front
Rear
ns ns
** ns
*** ns
*** ns
Nucleus
EMD RNAi
CTRL

Supplementary Figure 3 Distribution maps of chromosome territories.

a Distribution maps of chromosome 1, 9, 12, 18, 22 in control (left, P_{Chr12}=0.005, P_{Chr18}=0.0002, P_{Chr22}=0.0003, two-sided Kolmogorov-Smirnov test) and EMD knock-down (right, two-sided Cramer von Mises test). For each distribution map an exact number of cells from 3 independent experiments is stated in the figure. ***P<0.001 **P<0.01 *P<0.05, ns – not significant. **b** Representative images of single cells after FISH, control (left) and EMD knock-down (right), the magenta outline indicates nucleus border. **c** Chromosome territory distribution plots of normalized density, corresponding to maps in Supplementary Figure 3a. ***P<0.001 **P<0.01 *P<0.05, ns – not significant. For each distribution map an exact number of cells from 3 independent experiments is stated in the figure. Source data are provided as Source Data file.

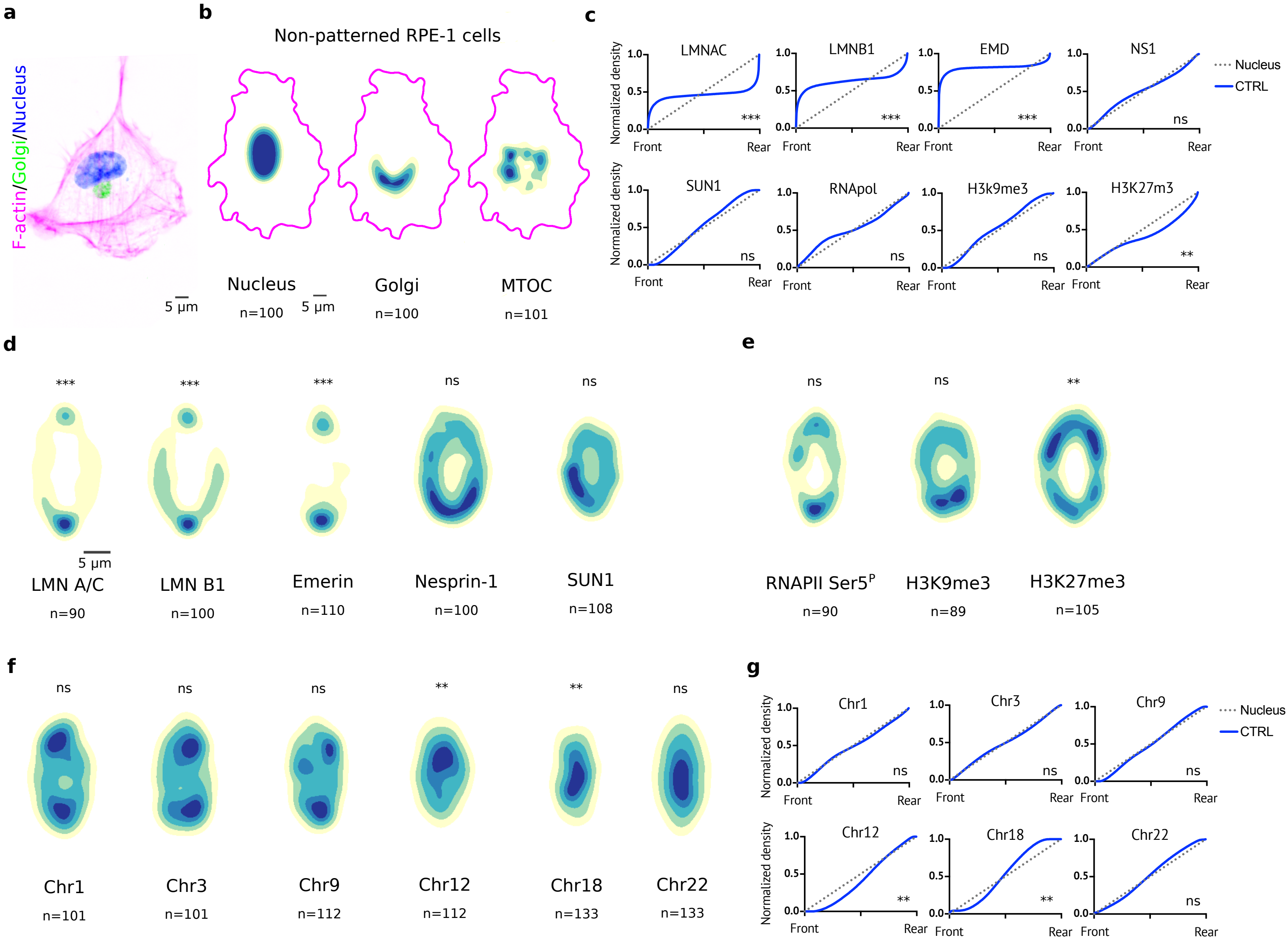

a
F-actin/Golgi/Nucleus
5 µm
b
Non-patterned RPE-1 cells
Nucleus
n=100
5 µm
Golgi
n=100
MTOC
n=101
c
LMNAC
LMNB1
EMD
NS1
SUN1
RNApol
H3k9me3
H3K27m3
Normalized density
Front
Rear
Nucleus
CTRL
***
ns
**
d
LMN A/C
n=90
LMN B1
n=100
Emerin
n=110
Nesprin-1
n=100
SUN1
n=108
e
RNAPII Ser5P
n=90
H3K9me3
n=89
H3K27me3
n=105
f
Chr1
n=101
Chr3
n=101
Chr9
n=112
Chr12
n=112
Chr18
n=133
Chr22
n=133
g
Chr1
Chr3
Chr9
Chr12
Chr18
Chr22

Supplementary Figure 4 Mapping front-rear polarity in the nucleus in non-patterned cells.

a Single non-patterned RPE-1 cell. **b** Distribution maps of nucleus, Golgi, and microtubule-organizing center (MTOC) in non-patterned RPE1 cells, outline represents F-actin border of the averaged cells. **c** Protein distribution plots of normalized density, corresponding to maps in Supplementary Figure 4d-e. ***P<0.001 **P<0.01 *P<0.05, ns – not significant. **d** Distribution maps of lamins (LMN), emerin and LINC-complex proteins. $P_{LMNAc}=9.6x10^{-12}$, $P_{LMNB1}=2.8x10^{-12}$, $P_{EMD}=4.7x10^{-8}$, two-sided Kolmogorov-Smirnov test, ***P<0.001 **P<0.01 *P<0.05, ns – not significant. **e** Distribution maps of transcription-related markers RNAPII $Ser5_P$ – Ser5 phosphorylated RNA polymerase II, H3K9me3 and H3K27me3. $P_{H3K27me3}=0.002$, two-sided Kolmogorov-Smirnov test, ***P<0.001 **P<0.01 *P<0.05, ns – not significant **f** Distribution map of chromosome 1, 3, 9, 12, 18 and 22. $P_{Chr12}=0.006$, $P_{Chr18}=0.001$, two-sided Kolmogorov-Smirnov test, ***P<0.001 **P<0.01 *P<0.05, ns – not significant **g** Chromosome distribution plots of normalized density, corresponding to maps in Supplementary Figure 4f. ***P<0.001 **P<0.01 *P<0.05, ns – not significant. For each distribution map an exact number of cells from 3 independent experiments is stated in the figure. Source data are provided as Source Data file.

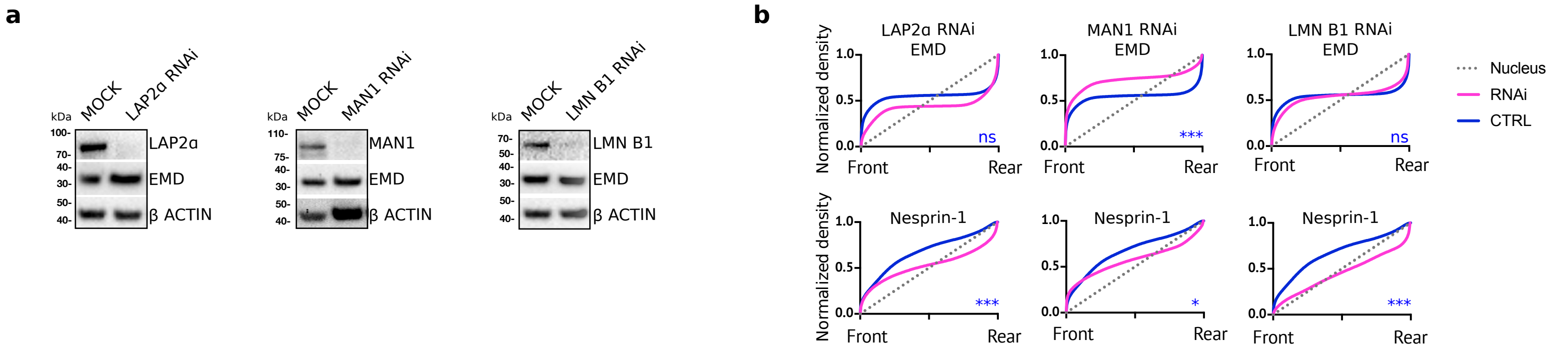
a
kDa
MOCK
LAP2α RNAi
100-
70-
40-
30-
50-
40-
LAP2α
EMD
β ACTIN
MAN1 RNAi
110-
75-
MAN1
LMN B1 RNAi
LMN B1
b
LAP2α RNAi
EMD
MAN1 RNAi
LMN B1 RNAi
Nesprin-1
Normalized density
1.0
0.5
0.0
Front
Rear
ns
***
*
Nucleus
RNAi
CTRL

Supplementary Figure 5 Effect of LAP2α, MAN1 and LMN B1 knock-down on nuclear polarity.

a Western blot of MOCK control and LAP2 α (left), MAN1 (middle) and LMNB1 (right) RNAi performed in RPE1 cells. **b** Normalized density plots of EMD and nesprin-1 distribution in LAP2 α (left), MAN1 (middle) and LMNB1 (right) RNAi, corresponding to maps in Figure 3a-c. ***P<0.001 **P<0.01 *P<0.05, ns – not significant. Source data are provided as Source Data file.

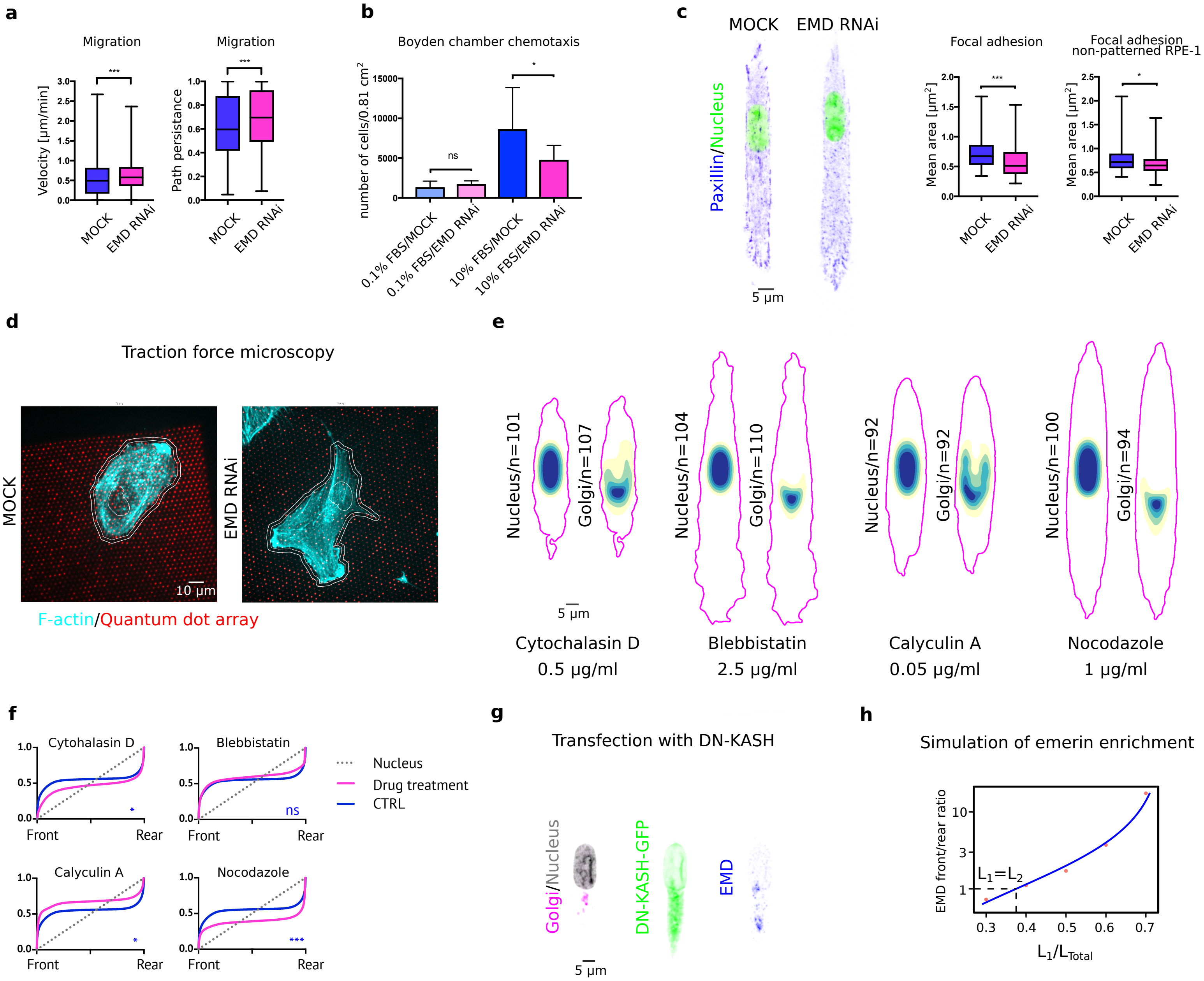
a
Migration
Velocity [µm/min]
MOCK
EMD RNAi
Migration
Path persistance
b
Boyden chamber chemotaxis
number of cells/0.81 cm²
0.1% FBS/MOCK
0.1% FBS/EMD RNAi
10% FBS/MOCK
10% FBS/EMD RNAi
c
MOCK
EMD RNAi
Paxillin/Nucleus
5 µm
Focal adhesion
Mean area [µm²]
Focal adhesion non-patterned RPE-1
d
Traction force microscopy
MOCK
EMD RNAi
10 µm
F-actin/Quantum dot array
e
Nucleus/n=101
Golgi/n=107
Nucleus/n=104
Golgi/n=110
Nucleus/n=92
Golgi/n=92
Nucleus/n=100
Golgi/n=94
5 µm
Cytochalasin D 0.5 µg/ml
Blebbistatin 2.5 µg/ml
Calyculin A 0.05 µg/ml
Nocodazole 1 µg/ml
f
Cytohalasin D
Blebbistatin
Calyculin A
Nocodazole
Front
Rear
Nucleus
Drug treatment
CTRL
g
Transfection with DN-KASH
Golgi/Nucleus
DN-KASH-GFP
EMD
5 µm
h
Simulation of emerin enrichment
EMD front/rear ratio
L1=L2
L1/LTotal

Supplementary Figure 6 Functional aspects of nuclear polarity.

a Quantification of migration parameters velocity and persistence n_{MOCK}=2666, $n_{EMDRNAi}$=2523 cells from 3 independent experiments, the plots was generated form 3 independent migration experiments. Both P values <$1x10^{-15}$. The boxes represent the mean values and the line in the box represents median. Whiskers represent the minimum and maximum values. Two-sided Kolmogorov-Smirnov test, ***P<0.001. **b** Quantification of chemotaxis efficiency using Boyden transwell assay, the plot was generated form 3 independent experiments, P=0.031. The boxes represent the mean values and the line in the box represents median. Whiskers represent the minimum and maximum values. Two-tailed Wilcoxon matched-pair test, *P<0.05. **c** Focal adhesion analysis, representative immunostaining images for paxillin for control and EMD RNAi. Quantification of mean area of focal adhesions for micro-patterned (n_{MOCK}=95, $n_{EMDRNAi}$=95 cells from 3 independent experiments), P=$0.1x10^{-5}$ and non-patterned cells (n_{MOCK}=97, $n_{EMDRNAi}$=99 cells from 3 independent experiments), P=0.011. The plots was generated form 3 independent experiments. The boxes represent the mean values and the line in the box represents median. Whiskers represent the minimum and maximum values. Two-sided Kolmogorov-Smirnov test, ***P<0.001, *P<0.05. **d** Representative images of control and EMD RNAi cells stained for F-actin (cyan) on quantum dot array (red) for traction force microscopy analysis. **e** Distribution maps of the nucleus and Golgi in cells treated with cytochalasin D, blebbistatin, calyculin A and nocodazole. For each distribution map an exact number of cells from 3 independent experiments is stated in the figure. **f** EMD and nesprin-1 distribution plots of normalized density in cells treated with cytochalasin D, blebbistatin, calyculin A and nocodazole, corresponding to maps in Figure 4f. P values in blue were calculated using two-sided Cramer von Mises test to compare distribution map of control cells and upon emerin knock-down. ***P<0.001 **P<0.01 *P<0.05, ns – not significant. **g** Single representative image of the cells transfected with dominant negative KASH domain-EGFP. **h** Both stochastic numerical simulation (red dots) and the analytical solution of the mathematical model (blue line) predict the same scaling of the EMD front-to-rear ratio at NE in function of the ER asymmetry, L_1 – ER frontal length, L_{Total} – total ER length. For each distribution map an exact number of cells from 3 independent experiments is stated in the figure. Source data are provided as Source Data file.

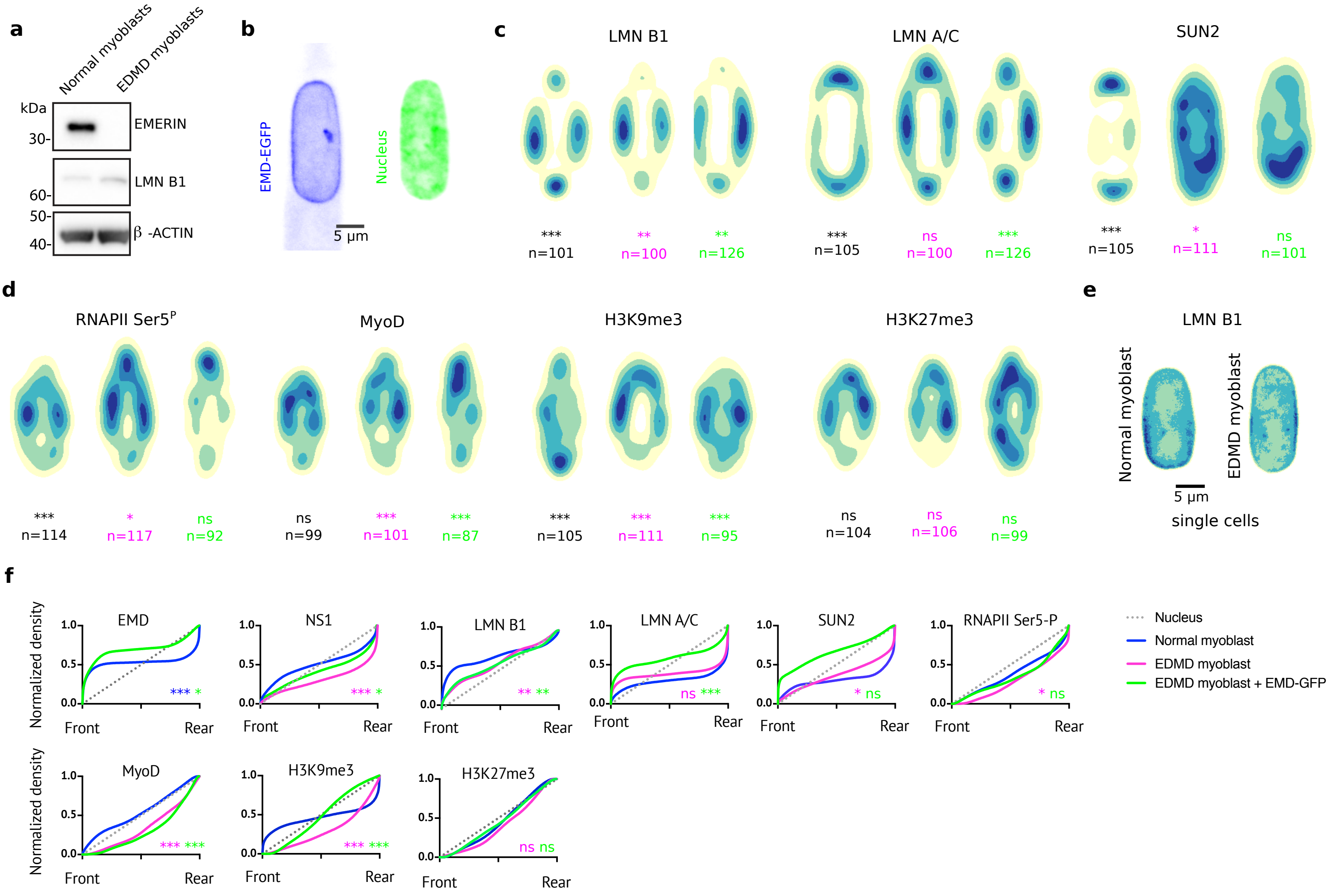
a
Normal myoblasts
EDMD myoblasts
kDa
30-
EMERIN
60-
LMN B1
50-
40-
β -ACTIN
b
EMD-EGFP
Nucleus
5 µm
c
LMN B1
LMN A/C
SUN2
***
n=101
**
n=100
**
n=126
***
n=105
ns
n=100
***
n=126
***
n=105
*
n=111
ns
n=101
d
RNAPII Ser5P
MyoD
H3K9me3
H3K27me3
***
n=114
*
n=117
ns
n=92
ns
n=99
***
n=101
***
n=87
***
n=105
***
n=111
***
n=95
ns
n=104
ns
n=106
ns
n=99
e
LMN B1
Normal myoblast
EDMD myoblast
5 µm
single cells
f
Normalized density
EMD
NS1
LMN B1
LMN A/C
SUN2
RNAPII Ser5-P
MyoD
H3K9me3
H3K27me3
1.0
0.5
0.0
Front
Rear
*** *
*** *
** **
ns ***
* ns
* ns
*** ***
*** ***
ns ns
Nucleus
Normal myoblast
EDMD myoblast
EDMD myoblast + EMD-GFP

Supplementary Figure 7 Nuclear front-rear polarity rescue in primary myoblasts.

a Western blot in primary normal and emerin-deficient myoblasts. **b** Representative image of localization of EMD-EGFP. **c** Distribution map of lamins (LMN) and SUN2 in normal (left, $P_{LMNB1}=4.6x10^{-15}$, $P_{LMNAC}=6.7x10^{-16}$, $P_{SUN2}=6.7x10^{-16}$, two-sided Kolmogorov-Smirnov test), EDMD (middle, $P_{LMNB1}=0.005$, $P_{SUN2}=0.003$, two-sided Cramer von Mises test) and EDMD+EMD-EGFP (right, $P_{LMNB1}=0.008$ $P_{LMNAC}=1.3x10^{-11}$, two-sided Cramer von Mises test) myoblasts. **d** Distribution map of transcription associated markers in normal (left, $P_{RNAPIISer5P}=0.0006$, $P_{H3K9me}=5.3x10^{-8}$, two-sided Kolmogorov-Smirnov test), EDMD (middle, $P_{RNAPIISer5P}=0.03$, $P_{MyoD}=6.5x10^{-6}$, $P_{H3K9me3}=1.2x10^{-6}$, two-sided Cramer von Mises test) and EDMD+EMD-EGFP (right, $P_{MyoD}=5.5x10^{-8}$, $P_{H3K9me3}=1.1x10^{-6}$, two-sided Cramer von Mises test) myoblasts. **e** Representative image of antibody staining for LMN B1 in normal and EDMD myoblasts. **f** Protein distribution plots of normalized density, corresponding to maps in Figure 5d-e and Supplementary Figure 7c-d. ***P<0.001 **P<0.01 *P<0.05, ns – not significant. For each distribution map an exact number of cells from 3 independent experiments is stated in the figure. Source data are provided as Source Data file.

Supplementary Text

In combination with the direct stochastic simulations described in the methods, we designed a minimal mathematical description representing the emerin transfer from the ER to the NE via a diffusive process (Supplementary Information Fig. 1**)** under the assumptions that (1) the rate of emerin appearance in the ER is proportional to the ER surface (due to surface-coupled synthesis and/or a surface-limited adsorption process), and (2) the emerin diffusion in the NE is slow enough to be neglected.

Since "transverse" diffusion along the ER does not affect he scaling of the mean rate of arrival into the NE by diffusion, the problem is effectively one-dimensional, and the average dynamics of N_{ER} and N_E (number of emerin molecules in the ER and in the NE, respectively) can be mathematically described by the following two-dimensional dynamical system (1):

$$\begin{cases} \acute{N}_{ER} = bL/L_{tot} - (d+k)N_{ER} \\ \acute{N}_E = kN_{ER} - dN_E \end{cases}$$

Where the dots represent time derivatives, b is the synthesis or appearance rate of emerin molecules on the ER surface per unit of surface, d their decay rate (assumed equal in ER and in the NE), k the rate of transfer from ER to NE due to diffusion, assumed to be due to a simple diffusion process, neglecting edge effects (2):

$$k = \tau^{-1} = \frac{2D}{L^2}$$

where D is the diffusion coefficient of emerin molecules in the ER.

The dynamical system can be solved, yielding (3,4):

$$N_{ER}(t) = N_{ER}(0)e^{-(d+k)t} + \frac{bL}{L_{tot}}\frac{1}{(d+k)}\left(1 - e^{-(d+k)t}\right),$$

$$N_E(t) = N_E(0)e^{-dt} + \frac{bL}{L_{tot}}\frac{k}{d(d+k)}(1 - e^{-dt}) + + \left[N_{ER}(0) - \frac{bL}{L_{tot}}\frac{1}{(d+k)}\right]e^{-dt}(1 - e^{-kt}).$$

We can also express the number of emerin molecules at steady state ($t \to +\infty$) in the ER and in the NE as a function of the parameters of the model (5,6):

$$N_{ER} = \frac{bL}{L_{tot}}\frac{1}{(d+k)},$$

$$N_E = \frac{bLk}{L_{tot}}\frac{1}{d(d+k)}.$$

The total number of emerin molecules at steady state is then (7):

$$N_{ER} + N_E = \frac{b}{d}\frac{L}{L_{tot}},$$

which provides a way to estimate the synthesis rate b by a measurement of emerin numbers/concentration. Finally,

the fraction of emerin molecules in the nuclear envelope is given by (8):

$$\frac{N_E}{N_{ER}+N_E}=\frac{k}{d+k}=\frac{1}{1+\frac{L^2 d}{2D}}.$$

We now consider the ER being interspaced by the NE (Fig. 4g; Supplementary Figure 6h), and suppose that the relative position between ER and NE may change, resulting in a differential volume of ER at the two opposite sides of the nucleus. We can at first consider the ER to have uniform width, so to treat its length $L_{tot}=L_1+L_2$ as a proxy for surface.

We are interested in studying the effect of the L_1/L_2 asymmetry on the emerin intake at the front and rear sides of the nuclear envelope, under the aforementioned hypotheses. We can thus compute the front/rear ratio in the number of emerin molecules which, at steady-state, reach the NE as a function of the front and back ER lengths L_1, L_2 (9):

$$\frac{N_E^{(1)}}{N_E^{(2)}}=\frac{L_1}{L_2}\frac{k_1}{k_2}\frac{(d+k_2)}{(d+k_1)}$$

which yields, since $k=2\,D/L^2$ (10),

$$\frac{N_E^{(1)}}{N_E^{(2)}}=\frac{L_2}{L_1}\frac{\left(1+\frac{2D}{L_2^2 d}\right)}{\left(1+\frac{2D}{L_1^2 d}\right)}.$$

At fixed L_1/L_2 ratios, we can obtain two different regimes depending on the k/d ratio (11):

$$\frac{N_E^{(1)}}{N_E^{(2)}}=\begin{cases}\frac{L_1}{L_2} & \text{if } \frac{2D}{L_{1,2}^2 d}\gg 1\\ \frac{L_2}{L_1} & \text{if } \frac{2D}{L_{1,2}^2 d}\ll 1\end{cases}.$$

To understand which one of the two regimes applies to our experimental system, we can estimate the $k/d=2\,D/L^2\,d$ ratio in the case of emerin diffusion in the ER. The emerin diffusion coefficient in ER can be approximated with that of GFP in ER ($D\simeq 10\,\mu m^2/s$)[1]. Emerin decay time under the considered experimental conditions was observed to be around $d^{-1}\simeq 50\,h$. The total length of the ER (and thus that of the front and rear fractions) is of the order of $100\,\mu m$. This yields (12):

$$\frac{k}{d}=\frac{2D}{L_{1,2}^2 d}\approx\frac{20\frac{\mu m^2}{s}}{10^4\mu m^2(50*3600s)^{-1}}\simeq 360\gg 1.$$

In other words, the estimates lead to the conclusion that in the case of emerin diffusion in ER we are in the “high” k/d regime, which yields (13):

$$\frac{N_E^{(1)}}{N_E^{(2)}} = \frac{L_1}{L_2}.$$

These analytical predictions of the mathematical model are in very good agreement with the direct stochastic simulations of the birth/death diffusion process (Fig. 4g; Supplementary Figure 6h).

In the model described so far we neglected all the possibility of active and directed processes that would transport emerin molecules towards the nucleus, but these processes may also be present. Their relevance will depend on the comparison between the diffusion characteristic time scale τ_{diff} and the time scales of active ("ballistic") transport τ_{ball} (14)

$$\begin{cases} \tau_{diff} = \dfrac{L^2}{2D} \\ \tau_{ball} = \dfrac{L}{v} \end{cases}$$

where v is the typical speed resulting from the action of molecular motors. In the "ballistic" limit case ($\tau_{ball} \ll \tau_{diff}$, $v \gg L/2\,D$), the characteristic time to reach the nuclear envelope scales as L rather than as L^2 (15):

$$\begin{cases} \acute{N}_{ER} = bL/L_{tot} - \left(d + \dfrac{v}{L}\right) N_{ER} \\ \acute{N}_E = \dfrac{v}{L} N_{ER} - dN_E \end{cases}.$$

The front/back ratio in the number of emerin molecules at steady-state has two different regimes (16):

$$\frac{N_E^{(1)}}{N_E^{(2)}} = \begin{cases} \dfrac{L_1}{L_2} & \text{if } \dfrac{v}{L_{1,2}d} \gg 1 \\ 1 & \text{if } \dfrac{v}{L_{1,2}d} \ll 1 \end{cases}.$$

Hence, as in the diffusive model the emerin front/back ratio at the two opposite sides of the nuclear envelope corresponds to the ER front/rear ratio when the endocytosis velocity is not too high with respect to the decay rate of the molecule ($v/L \gg d$). Thus, in this regime, the two models are not distinguishable. On the contrary, the prediction of the ballistic case differs from that of the diffusive case in the regime of high decay / low velocity ($v/L \ll d$), where the former would predict an equal distribution of emerin at the two sides of the nuclear envelope irrespective of L_1, L_2.

Finally, we considered the case where the assumption of emerin synthesis/adsorption proportional to the ER surface is relaxed. In this case, the scaling of the front/rear emerin ratio is not compatible with the experimental observations. Indeed, the ODEs of this variant of the model, reading (17):

$$\begin{cases} \acute{N}_{ER} = b - (d + k)N_{ER} \\ \acute{N}_E = kN_{ER} - dN_E \end{cases},$$

yield to the following steady-state values for the emerin molecules at the NE (18):

$$N_E = \frac{bk}{d(d+k)}.$$

The front/rear ratio in the number of emerin molecules which, at steady-state, reach the NE as a function of the front and back ER lengths L_1, L_2 is then (19):

$$\frac{N_E^{(1)}}{N_E^{(2)}} = \frac{L_2}{L_1}\frac{\left(1+\frac{2D}{L_2^2 d}\right)}{\left(1+\frac{2D}{L_1^2 d}\right)}.$$

Again, at fixed L_1/L_2 ratios, we can obtain two different regimes depending on the k/d ratio (20):

$$\frac{N_E^{(1)}}{N_E^{(2)}} = \begin{cases} 1 \quad \text{if} \quad \frac{2D}{L_{1,2}^2 d} \gg 1 \\ \frac{L_2^2}{L_1^2} \text{if} \quad \frac{2D}{L_{1,2}^2 d} \ll 1 \end{cases}.$$

In other words, the front-rear steady-state ratio in the number of emerin at the NE is never proportional to the length ratio (as observed experimentally) in this model variant where emerin synthesis/adsorption is not proportional to ER surface.

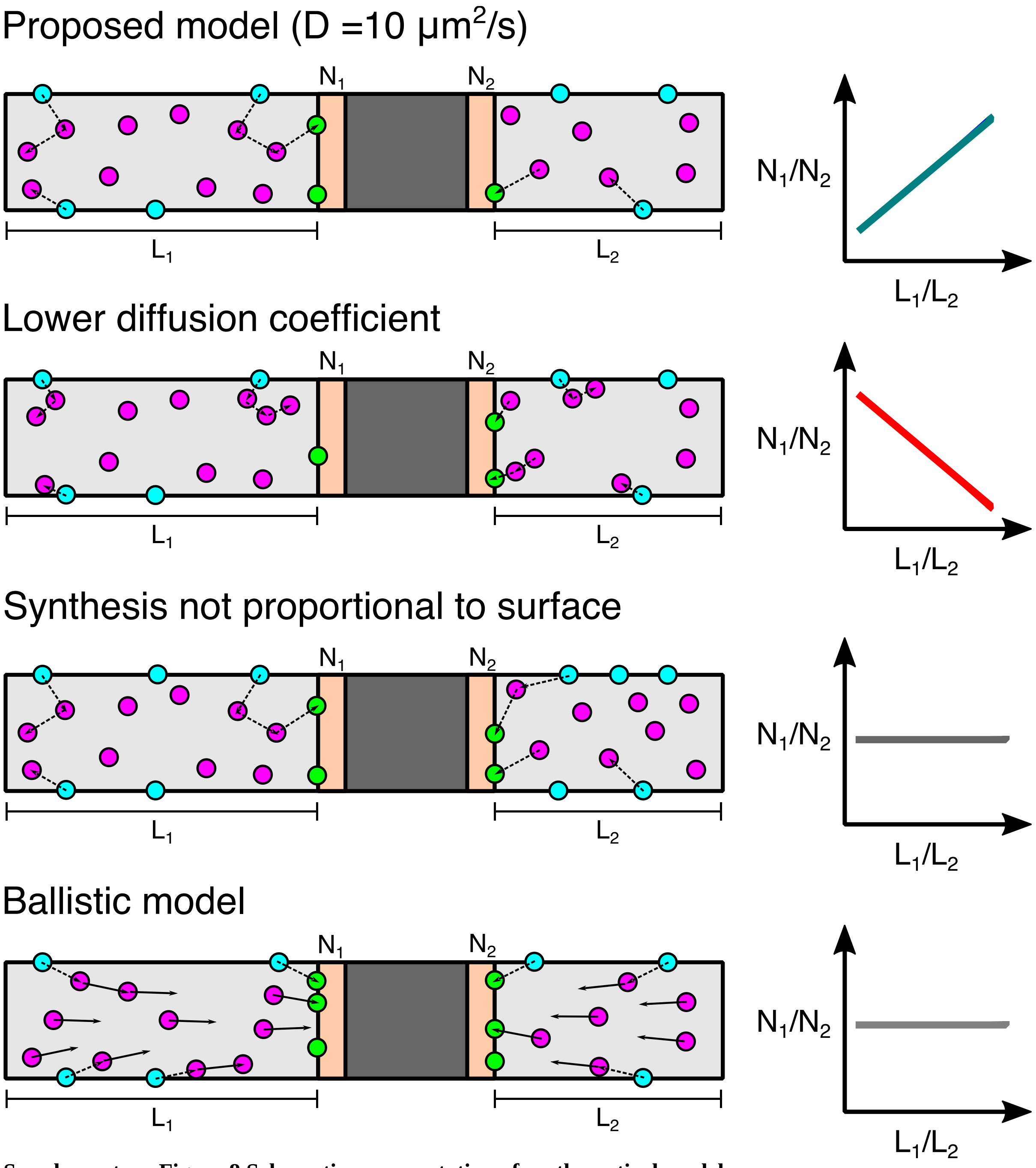

Supplementary Figure 8 Schematic representation of mathematical model

Variants of the proposed mathematical model, based on different assumptions, yield different emerin front-to-back ratios. **a** Our current model predicts the emerin front-to-back ratio to scale linearly with the ER front-to-back ratio. **b** A much lower diffusivity (at least 5 orders of magnitude than that of emerin) would cause the front-to-back ratio to be negatively proportional to the amount of ER present on each side. **c-d** Finally, by relaxing the assumption that EMD synthesis is proportional to the ER surface, or by considering a ballistic model (where we considered endocytic processes that dragging emerin molecules towards the nucleus) would result in a front-to-back ratio equal to 1. **Color legend**: light blue circles: newly synthesized EMD; purple circles: EMD free in the ER; green circles: EMD reaching the nuclear envelope.

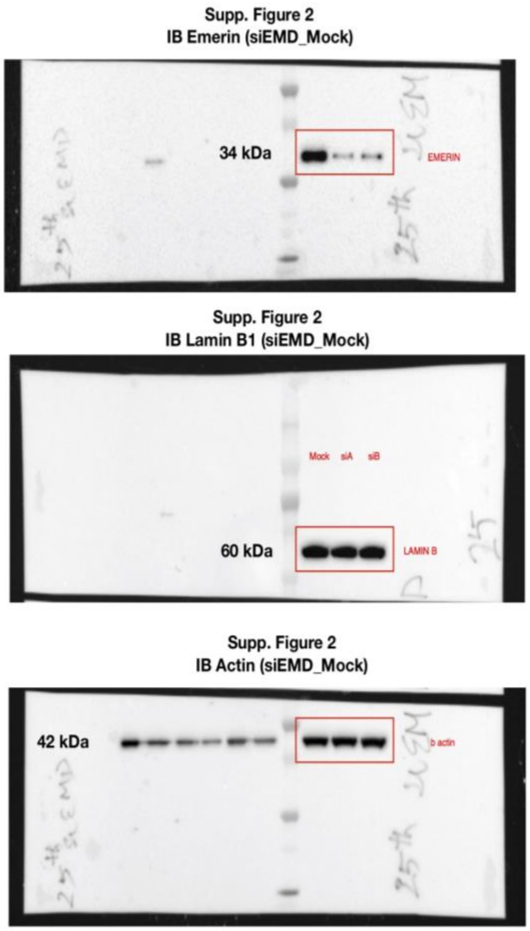

Supplementary Figure 9 Uncropped immunoblots from Supplementary Figure 2

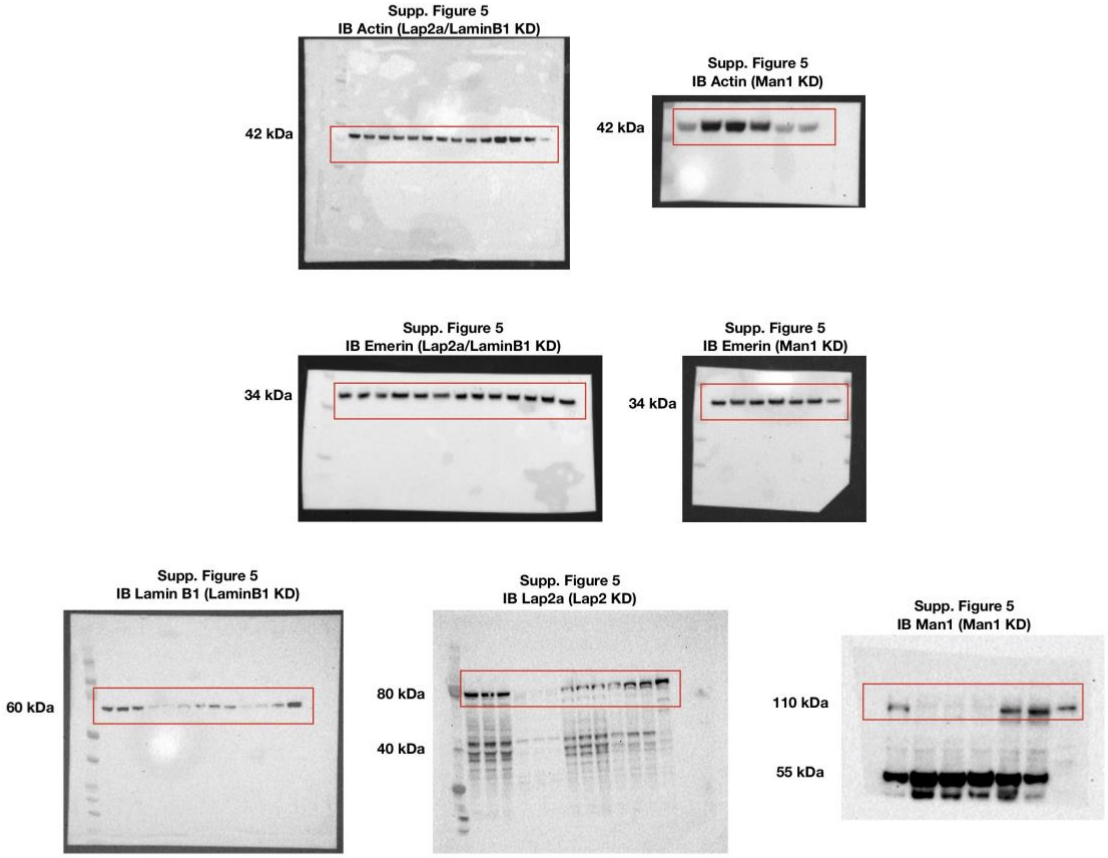

Supplementary Figure 10 Uncropped immunoblots from Figure 5

Supp. Figure 7A
IB Emerin (siEMD_EDMD_Ctrl)

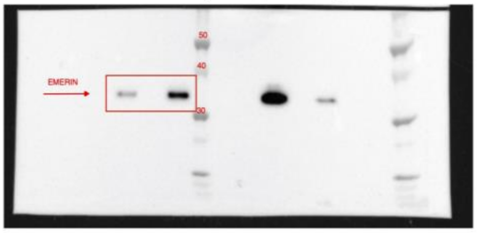

Supp. Figure 7A
IB Lamin B1 (siEMD_EDMD_Ctrl)

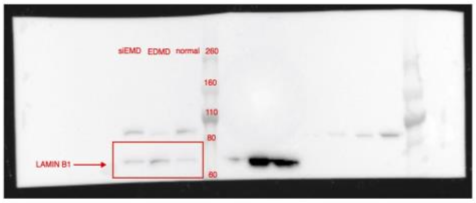

Supp. Figure 7A
IB Actin (siEMD_EDMD_Ctrl)

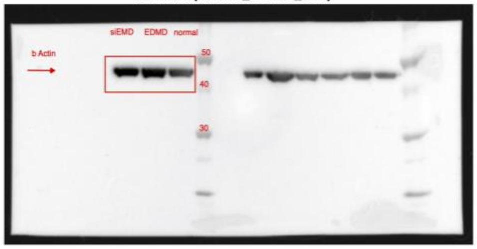

Supplementary Figure 11 Uncropped immunoblots from Supplementary Figure 7

Supplementary Table 1.

List of primary antibodies used in the study.

Antibody	Clone	Clonality	Catalog #	Host	Provider	Dilution
α-Giantin [Golgi marker]	PRB-114C	Polyclonal	PRB-114C	rabbit	Biolegend	1:1000
α-Giantin [Golgi marker]	PRB-114C, Alexa Fluor 488-conjugated	Polyclonal	PRB-114C	rabbit	Biolegend	1:1000
BANF1	EPR7668	Monoclonal	ab129184	rabbit	Abcam	1:400
CENPB [Centromere marker]	-	Polyclonal	ab25734	rabbit	Abcam	1:500
Emerin	4G5	Monoclonal	NCL-EMERIN	mouse	Leica Biosystems	1:500
Emerin	H-12	Monoclonal	Sc-25284	Mouse	Santa Cruz Biotech	1:300
F-Actin	-	-	-	-	Sigma	1:100
H3K27me3	-	Monoclonal	C36B11	rabbit	Cell Signaling Technology	1:1600
H3K4me3	-	Polyclonal	39159	rabbit	Active Motif	1:1000
H3K9ac	-	Polyclonal	07-352	rabbit	Milipore	1:300
H3K9me3	-	Polyclonal	ab8898	rabbit	Abcam	1:500
KDEL [ER marker]	EPR12668	Monoclonal	ab176333	rabbit	Abcam	1:250
Ki67	SP6	Monoclonal	ab16667	rabbit	Abcam	1:250
Lamin A/C	636	Monoclonal	sc7292	mouse	Santa Cruz Biotech	1:500
Lamin A/C (Phospho Ser22)	-	Monoclonal	D2B2E	rabbit	Cell Signaling Technology	1:2500
Lamin B1	-	Polyclonal	ab16048	rabbit	Abcam	1:500
LAP2 α	-	Polyclonal	ab5162	rabbit	Abcam	1:400
MAN1	-	Polyclonal	ab121854	rabbit	Abcam	1:400
MyoD	-	Monoclonal	sc-377460	mouse	Santa Cruz Biotech	1:300
Nesprin 1	MANNES1A (7A12)	Monoclonal	MA5-18077	mouse	Thermo Scientific	1:200
Nesprin 1	-	Polyclonal	HPA019113	rabbit	Sigma Life Science	1:300
Nesprin 2	K20-478-5	Monoclonal	K20-478-5	mouse	Thermo Scientific	1:500
Nucleolin [Nucleolus marker]	-	Polyclonal	ab22758	rabbit	Abcam	1:800
Paxillin	Y113	Monoclonal	ab32084	Rabbit	Abcam	1: 600
Pericentrin	mAbcam 28144	Monoclonal	ab28144	mouse	Abcam	1: 500
RNA Polymerase II (Phospho Ser5)	-	Polyclonal	ab5131	rabbit	Abcam	1: 200
SUN1	EPR6554	Monoclonal	ab124770	rabbit	Abcam	1:500
SUN2	EPR6557	Monoclonal	ab124916	rabbit	Abcam	1:400
TRF2 [Telomere marker]	4A794	Monoclonal	05-521	mouse	Milipore	1:200

Vinculin	hVIN-1	Monoclonal	V9131	mouse	Sigma	1:400
B-actin	AC-15	Monoclonal	ab6276	mouse	Abcam	1:200

References

1. Dayel, M. J., Horn, E. F. Y. & Verkman, A. S. Diffusion of green fluorescent protein in the aqueous-phase lumen of endoplasmic reticulum. *Biophys. J.* **76**, 2843–2851 (1999).
