# Supplementary material for: Role of the nuclear membrane protein Emerin in front-rear polarity of the nucleus
Source: Nat Commun. 2020 May 1;11:2122. doi: 10.1038/s41467-020-15910-9 (PMC7195445; doi:10.1038/s41467-020-15910-9)
Supplement: Supplementary file 3 — Description of Additional Supplementary Files [file 41467_2020_15910_MOESM3_ESM.pdf]

Description of Additional Supplementary Files

File Name: Supplementary Movie 1
Description: Live cell imaging of emerin-EGFP. Representative RPE1 EMD-EGFP expressing cell migrating on micro-patterned lines visualized using DIC and color-coded NE analysis of EMD-EGFP. Time in hours, scale bar 10 µm.

File Name: Supplementary Movie 2
Description: Simulation of emerin enrichment at the nuclear envelope.
