# Supplementary material for: Role of the nuclear membrane protein Emerin in front-rear polarity of the nucleus
Source: Nat Commun. 2020 May 1;11:2122. doi: 10.1038/s41467-020-15910-9 (PMC7195445; doi:10.1038/s41467-020-15910-9)
Supplement: Supplementary file 6 — Reporting Summary [file 41467_2020_15910_MOESM6_ESM.pdf]

nature research

Corresponding author(s): Paolo Maiuri

Last updated by author(s): Jan 9, 2020

Reporting Summary

Nature Research wishes to improve the reproducibility of the work that we publish. This form provides structure for consistency and transparency in reporting. For further information on Nature Research policies, see Authors & Referees and the Editorial Policy Checklist .

Statistics

For all statistical analyses, confirm that the following items are present in the figure legend, table legend, main text, or Methods section.

n/a	Confirmed	
☐	☒	The exact sample size (*n*) for each experimental group/condition, given as a discrete number and unit of measurement
☐	☒	A statement on whether measurements were taken from distinct samples or whether the same sample was measured repeatedly
☐	☒	The statistical test(s) used AND whether they are one- or two-sided<br>*Only common tests should be described solely by name; describe more complex techniques in the Methods section.*
☒	☐	A description of all covariates tested
☐	☒	A description of any assumptions or corrections, such as tests of normality and adjustment for multiple comparisons
☐	☒	A full description of the statistical parameters including central tendency (e.g. means) or other basic estimates (e.g. regression coefficient) AND variation (e.g. standard deviation) or associated estimates of uncertainty (e.g. confidence intervals)
☐	☒	For null hypothesis testing, the test statistic (e.g. *F*, *t*, *r*) with confidence intervals, effect sizes, degrees of freedom and *P* value noted<br>*Give P values as exact values whenever suitable.*
☒	☐	For Bayesian analysis, information on the choice of priors and Markov chain Monte Carlo settings
☒	☐	For hierarchical and complex designs, identification of the appropriate level for tests and full reporting of outcomes
☒	☐	Estimates of effect sizes (e.g. Cohen's *d*, Pearson's *r*), indicating how they were calculated

Our web collection on statistics for biologists contains articles on many of the points above.

Software and code

Policy information about availability of computer code

Data collection	Velocity software PerkinElmer (version 6.3)
Data analysis	R studio (http://www.r-project.org/) , custom-built ImageJ (version 1.52i) plugins, GraphPad Prism (version 7.0 d)

For manuscripts utilizing custom algorithms or software that are central to the research but not yet described in published literature, software must be made available to editors/reviewers. We strongly encourage code deposition in a community repository (e.g. GitHub). See the Nature Research guidelines for submitting code & software for further information.

Data

Policy information about availability of data

All manuscripts must include a data availability statement. This statement should provide the following information, where applicable:

- Accession codes, unique identifiers, or web links for publicly available datasets
- A list of figures that have associated raw data
- A description of any restrictions on data availability

The authors declare that all data supporting the findings of this study are available within the article and its Supplementary Information files or from the corresponding author upon reasonable request.

Field-specific reporting

Please select the one below that is the best fit for your research. If you are not sure, read the appropriate sections before making your selection.

☒ Life sciences ☐ Behavioural & social sciences ☐ Ecological, evolutionary & environmental sciences

October 2018

Life sciences study design

All studies must disclose on these points even when the disclosure is negative.

Sample size	Sufficient replicates for statistical significance, at least three and often more were undertaken. The sample size is indicated for each experiment.
Data exclusions	No data exclusions were necessary in this study.
Replication	Data was successfully replicated in at least 3 independent experiments and can be reproduced.
Randomization	Cells were randomly assigned to this study. For distribution map preparation we performed sample randomization.
Blinding	Blinding was not possible for our experiments. However,all imaging samples were quantified using the least biased approach possible. This included the use of image analysis programs as indicated in the Methods section.

Reporting for specific materials, systems and methods

We require information from authors about some types of materials, experimental systems and methods used in many studies. Here, indicate whether each material, system or method listed is relevant to your study. If you are not sure if a list item applies to your research, read the appropriate section before selecting a response.

Materials & experimental systems

n/a	Involved in the study	
☐	☒	Antibodies
☐	☒	Eukaryotic cell lines
☒	☐	Palaeontology
☒	☐	Animals and other organisms
☒	☐	Human research participants
☒	☐	Clinical data

Methods

n/a	Involved in the study	
☒	☐	ChIP-seq
☒	☐	Flow cytometry
☒	☐	MRI-based neuroimaging

Antibodies

Antibodies used

α-Giantin [Golgi marker] PRB-114C Polyclonal PRB-114C rabbit Biolegend
α-Giantin [Golgi marker] PRB-114C, Alexa Fluor 488-conjugated Polyclonal PRB-114C rabbit Biolegend
BANF1 EPR7668 Monoclonal ab129184 rabbit Abcam
CENPB [Centromere marker] - Polyclonal ab25734 rabbit Abcam
Emerin 4G5 Monoclonal NCL-EMERIN mouse Leica Biosystems
Emerin H-12 Monoclonal Sc-25284 Mouse Santa Cruz Biotech
H3K27me3 - Monoclonal C36B11 rabbit Cell Signaling Technology
H3K4me3 - Polyclonal 39159 rabbit Active Motif
H3K9ac - Polyclonal 07-352 rabbit Milipore
H3K9me3 - Polyclonal ab8898 rabbit Abcam
KDEL [ER marker] EPR12668 Monoclonal ab176333 rabbit Abcam
Ki67 SP6 Monoclonal ab16667 rabbit Abcam
Lamin A/C 636 Monoclonal sc7292 mouse Santa Cruz Biotech
Lamin A/C (Phospho Ser22) - Monoclonal D2B2E rabbit Cell Signaling Technology
Lamin B1 - Polyclonal ab16048 rabbit Abcam
LAP2 α - Polyclonal ab5162 rabbit Abcam
MAN1 - Polyclonal ab121854 rabbit Abcam
MyoD - Monoclonal sc-377460 mouse Santa Cruz Biotech
Nesprin 1 MANNES1A(7A12) Monoclonal MA5-18077 mouse Thermo Scientific
Nesprin 1 - Polyclonal HPA019113 rabbit Sigma Life Science
Nesprin 2 K20-478-5 Monoclonal K20-478-5 mouse Thermo Scientific
Nucleolin [Nucleolus marker] - Polyclonal ab22758 rabbit Abcam
Paxillin Y113 Monoclonal ab32084 Rabbit Abcam
Pericentrin mAbcam 28144 Monoclonal ab28144 mouse Abcam
RNA Polymerase II (Phospho Ser5) - Polyclonal ab5131 rabbit Abcam
SUN1 EPR6554 Monoclonal ab124770 rabbit Abcam
SUN2 EPR6557 Monoclonal ab124916 rabbit Abcam
TRF2 [Telomere marker] 4A794 Monoclonal 05-521 mouse Milipore

Vinculin hVIN-1 Monoclonal V9131 mouse Sigma
B-actin AC-15 Monoclonal ab6276 mouse Abcam

Dilutions of the antibodies are provided in Supplementary Table 1

F-actin was visualized using Phalloidin, Fluorescein Isothiocyanate Labeled (Sigma-Aldrich)

Secondary antibodies:
Polyclonal Donkey anti-mouse AlexaFluor-488 AB_2340846 (Jackson ImmunoResearch)
Polyclonal Donkey anti-mouse AlexaFluor-594 AB_2340854 (Jackson ImmunoResearch)
Polyclonal Donkey anti-mouse AlexaFluor-Cy3 AB_2340813 (Jackson ImmunoResearch)
Polyclonal Donkey anti-rabbit AlexaFluor-488 AB_2313584 (Jackson ImmunoResearch)
Polyclonal Donkey anti-rabbit AlexaFluor-594 AB_2340621 (Jackson ImmunoResearch)
Polyclonal Donkey anti-rabbit AlexaFluor-Cy3 AB_2307443 (Jackson ImmunoResearch)

Validation

Validation was according to manufacturers instructions on their websites, please find links below:

α-Giantin [Golgi marker] PRB-114C and α-Giantin-Alexa Fluor 488-conjugated Polyclonal:
https://www.biolegend.com/en-us/reproducibility

BANF1 EPR7668 Monoclonal ab129184 rabbit Abcam
CENPB [Centromere marker] - Polyclonal ab25734 rabbit Abcam
HH3K9me3 - Polyclonal ab8898,
KDEL [ER marker] EPR12668 Monoclonal ab176333 rabbit Abcam
Ki67 SP6 Monoclonal ab16667 rabbit Abcam
Lamin B1 - Polyclonal ab16048 rabbit Abcam
LAP2 α - Polyclonal ab5162 rabbit Abcam
MAN1 - Polyclonal ab121854 rabbit Abcam
Nucleolin [Nucleolus marker] - Polyclonal ab22758 rabbit Abcam
Paxillin Y113 Monoclonal ab32084 Rabbit Abcam
Pericentrin mAbcam 28144 Monoclonal ab28144 mouse Abcam
RNA Polymerase II (Phospho Ser5) - Polyclonal ab5131 rabbit Abcam
SUN1 EPR6554 Monoclonal ab124770 rabbit Abcam
SUN2 EPR6557 Monoclonal ab124916 rabbit Abcam
B-actin AC-15 Monoclonal ab6276 mouse Abcam
https://www.abcam.com/primary-antibodies/a-guide-to-antibody-validation

Emerin 4G5 Monoclonal NCL-EMERIN mouse Leica Biosystems
Technical pdf sheet on the website: https://shop.leicabiosystems.com/us/ihc-ish/ihc-primary-antibodies/pid-emerin

H3K27me3 - Monoclonal C36B11 rabbit Cell Signaling Technology
Lamin A/C (Phospho Ser22) - Monoclonal D2B2E rabbit Cell Signaling Technology
https://www.cellsignal.com/contents/our-approach-antibody-validation-principles/antibody-validation-for-immunofluorescence/ourapproach-validation-if

Emerin H-12 Monoclonal Sc-25284 Mouse Santa Cruz Biotech
MyoD - Monoclonal sc-377460 mouse Santa Cruz Biotech
https://www.scbt.com/resources/protocols/immunofluorescence-cell-staining

H3K4me3 - Polyclonal 39159 rabbit Active Motif
https://www.activemotif.com/antibody-development

Nesprin 1 MANNES1A(7A12) Monoclonal MA5-18077 mouse Thermo Scientific
Nesprin 2 K20-478-5 Monoclonal K20-478-5 mouse Thermo Scientific
https://www.thermofisher.com/content/dam/LifeTech/Documents/PDFs/PG1705-PJT2509-COL05292-RO-Ab-Validation-Wiley-MiniBook-Americas-Final.pdf

H3K9ac - Polyclonal 07-352 rabbit Milipore
TRF2 [Telomere marker] 4A794 Monoclonal 05-521 mouse Milipore
Vinculin hVIN-1 Monoclonal V9131 mouse Sigma
Nesprin 1 - Polyclonal HPA019113 rabbit Sigma Life Science

https://www.sigmaaldrich.com/technical-documents/articles/biology/antibody-enhanced-validation.html

Eukaryotic cell lines

Policy information about cell lines

Cell line source(s)	Human hTERT-immortalized RPE-1 cell line was purchased from American Type Culture Collection (ATCC). Human primary myoblasts were obtained from Telethon Biobank including normal muscle biopsy (male, catalog#70515) and patient with EDMD (male, catalog# 49031, Emd mutation cDNA.539_543delTCTAC).
Authentication	Cell line authentication of human hTERT RPE-1 cell line was regularly performed by genetic profiling using polymorphic short tandem repeat (STR) loci. Human primary myoblasts were not authenticated.
Mycoplasma contamination	Mycoplasma testing was regularly conducted to assure that all cells used were mycoplasma free at all times.
Commonly misidentified lines (See ICLAC register)	No commonly misidentified lines were used in this study.
